# Transplantation strategy affects the risk of GvHD after prophylactic and preemptive donor lymphocyte infusion

**DOI:** 10.1007/s00277-025-06662-x

**Published:** 2025-10-20

**Authors:** Eva A.S. Koster, Peter A. von dem Borne, Joost G.K. van der Hem, Peter van Balen, Erik W.A. Marijt, Jennifer M.L. Tjon, Tjeerd J.F. Snijders, Daniëlle van Lammeren, Hendrik Veelken, J.H. Frederik Falkenburg, Liesbeth C. de Wreede, Constantijn J.M. Halkes

**Affiliations:** 1 Department of Hematology, Leiden University Medical Center, Leiden, The Netherlands; 2 Department of Hematology, Medical Spectrum Twente, Enschede, The Netherlands; 3 Department of Hematology, HagaZiekenhuis, The Hague, The Netherlands; 4 Department of Biomedical Data Sciences, Leiden University Medical Center, Leiden, The Netherlands

**Keywords:** Allogeneic stem cell transplantation, Donor lymphocyte infusion, Acute leukemia, Graft-versus-Host-Disease, Graft-versus-Leukemia effect, Chimerism

## Abstract

Donor lymphocyte infusion (DLI) after allogeneic stem cell transplantation (alloSCT) can boost Graft-versus-Leukaemia (GvL) reactivity but may induce Graft-versus-Host-Disease (GvHD). It is essential to understand which factors besides timing, donor type, and dose influence DLI alloreactivity. We previously identified viral infections, ≥ 5% patient cells in bone marrow chimerism, and lymphopenia at the time of DLI as relevant factors for GvHD after DLI following alemtuzumab-based T-cell depletion. Here, we investigated these factors and the alloreactivity after DLI following alloSCT with posttransplant cyclophosphamide in 83 patients with acute leukaemia/myelodysplastic syndrome receiving a prophylactic or preemptive DLI. 5% had viral infections close to DLI, 6% had ≥ 5% mixed chimerism, and 17% had lymphopenia. 2-year cumulative incidence of GvHD requiring systemic treatment was low: 7% (95%-confidence interval 1–14%). 22 of the 28 patients with ≥ 1% mixed chimerism at the time of DLI (79%) converted to full-donor chimerism. None of these responders relapsed, indicating achievement of GvL despite the low incidence of GvHD. Our data show that DLI alloreactivity is determined by the conditions at the time of DLI which are influenced by the transplantation strategy. Adjusting the DLI dose based on these conditions may improve the balance between GvHD and GvL.

**Supplementary Information:**

The online version contains supplementary material available at 10.1007/s00277-025-06662-x.

## Introduction

Relapse remains an important cause of failure of allogeneic stem cell transplantation (alloSCT) in patients with acute leukaemia. Unmodified donor lymphocyte infusions (DLI) can be given to boost the Graft-versus-Leukaemia (GvL) effect to prevent relapse, but may induce Graft-versus-Host-Disease (GvHD). To improve the balance between GvHD and GvL and thereby the applicability of DLI, it is crucial to better understand which factors influence the alloreactivity of DLI.

Expert opinion recommends that dosing of prophylactic and preemptive DLI should at least be based on donor type and time after alloSCT to reduce the risk of severe GvHD [1]. In a recent study, we identified three other risk factors for the development of GvHD after DLI following alemtuzumab-based T-cell depleted (TCD) alloSCT: occurrence of viral infections (*de novo* or reactivation) close to DLI, presence of patient-derived antigen-presenting cells (APCs) in the bone marrow (BM), and lymphopenia [2]. Patient-derived APCs are highly capable of activating donor-derived alloreactive T cells [3]. After alloSCT, the professional APCs of the patient are gradually replaced by donor-derived APCs. We previously showed that the replacement of APCs in the skin occurs predominantly between 3 and 6 months after alloSCT [4]. Thus, from 6 months onwards the BM chimerism status should be a good indicator of the origin of the professional APCs in the peripheral tissues. Viral infections and lymphopenia promote the activation of (alloreactive) T cells [5–7]. The presence of these factors at the time of DLI depends on the transplantation strategy (i.e., conditioning intensity, use and type of TCD, and GvHD prophylaxis). Posttransplant cyclophosphamide (PTCY) preferentially targets activated alloreactive T cells and favours recovery of regulatory T cells [8, 9]. This leads to relatively early lymphocyte recovery and better protection against severe infections compared to other TCD strategies [10–12]. Additionally, most patients achieve full-donor chimerism (FDC, < 1% patient cells) within two months after PTCY alloSCT [13]. This profile could therefore be associated with low alloreactivity of DLI following PTCY alloSCT [2]. Indeed, the risk of GvHD appears to be similar between haploidentical DLI following PTCY alloSCT and DLI from HLA-matched donors after non-PTCY alloSCT despite the larger genetic disparity [14]. In the non-haploidentical PTCY setting, only two studies have reported outcomes after DLI. Carnevale-Schianca et al. investigated 14 patients receiving therapeutic DLI after which none developed grade III-IV acute GvHD and 1 patient developed chronic GvHD [15]. They reported an overall response rate of 57%. However, as more than half of the patients also received systemic therapy or radiotherapy, the contribution of the DLI itself on disease control is unclear [15]. Shanmugasundaram et al. investigated 38 DLIs given to 21 patients after PTCY, of whom 8 with a non-haploidentical donor, and observed low risks of acute (8%) and chronic (3%) GvHD but limited efficacy with 11% and 15% complete response after DLI for relapse and mixed chimerism, respectively [16]. These reported risks of GvHD are considerably lower than those observed after non-haploidentical DLI following other transplantation strategies [2, 17, 18]. However, both studies involved a wide variety of conditioning regimens and DLI settings (i.e., timing since alloSCT, DLI dose and pre-DLI treatments such as chemotherapy and steroids), making it hard to investigate the impact of the transplantation strategy and DLI circumstances on the alloreactivity of DLI.

In the current study, we investigated DLI after non-haploidentical PTCY alloSCT in a more homogeneous cohort treated according to a standardized DLI protocol: all patients were scheduled for prophylactic DLI at 4 or 6 months after alloSCT with fixed doses based on timing and donor type. We analysed the conditions at the time of DLI and assessed the alloreactivity after DLI, i.e. development of clinically relevant GvHD, conversion of mixed chimerism (MC, ≥ 1% patient cells) to FDC and the risk of relapse. By following the same systematic approach we used in the setting of DLI after alemtuzumab-based TCD alloSCT, the impact of the transplantation strategy on the DLI conditions and alloreactivity can be investigated.

## Methods

### Study population

This observational study included all adult patients with acute myeloid leukaemia (AML), acute lymphoblastic leukaemia (ALL) or myelodysplastic syndrome with excess blasts (MDS-EB2) in complete morphologic remission who received PTCY alloSCT from a ≥ 8/10 HLA-matched donor at Leiden University Medical Center (LUMC, Leiden, The Netherlands) between April 2020 and December 2022. The DLI cohort consisted of all patients who received a first DLI scheduled at 4 or 6 months after alloSCT (actually administered at 3.7–5.2 months and 5.3–9.0 months, respectively) without prior relapse or therapeutic systemic immunosuppression (tIS) for GvHD. The study was approved by the Medical Ethics Committee Leiden The Hague Delft (RP 22.002). All patients signed informed consent for data collection and analysis. Data were analysed as of March 2024.

### Transplantation and DLI protocol

Myeloablative conditioning consisted either of cyclophosphamide (2 days 60 mg/kg iv) and total body irradiation (3 days 2 × 2 Gy), or of thiotepa (2 days 5 mg/kg iv), fludarabine (3 days 50 mg/m^2^ iv) and busulfan (3 days 4 × 0.8 mg/kg iv). Reduced-intensity conditioning consisted of fludarabine (5 days 30 mg/m^2^ iv), cyclophosphamide (2 days 14.5 mg/kg iv) and total body irradiation (1 day 2 Gy). All patients received 40 mg/kg cyclophosphamide intravenously on days + 3 and + 4, 3 × 15 mg/kg mycophenolate from day + 5 until + 28, and tacrolimus titrated at 5–10 ng/ml from day + 5 until + 84, after which it was tapered with the aim to stop by day + 120 or + 150, depending on the timing of the first scheduled DLI (i.e., at 4 or 6 months, respectively). Patients had to be off GvHD prophylaxis for at least 2 weeks before a DLI could be administered. Four CMV seropositive patients with a CMV negative donor who were transplanted after October 2021 received letermovir prophylaxis.

In the absence of GvHD requiring tIS, patients considered to have a high risk of early relapse were scheduled to receive a 4-month DLI (0.3 × 10^6^ or 0.15 × 10^6^ T cells/kg in case of related donor [RD] or unrelated donor [UD], respectively). Reasons for prophylactic 4-month DLI were high-risk disease characteristics or incomplete pretransplant treatment. Preemptive 4-month DLI was given if minimal residual disease (MRD) was present at 2 months after alloSCT or in case of rapidly increasing MC between 2 and 4 months after alloSCT. All patients without GvHD requiring tIS, including those who had received a 4-month DLI, were scheduled to receive a prophylactic 6-month DLI, i.e., regardless of their anticipated relapse risk and chimerism or MRD status (3 × 10^6^ or 1.5 × 10^6^ T cells/kg, respectively). None of the patients received GvHD prophylaxis after DLI. Patients with persisting or increasing MC or MRD after the 6-month DLI could receive additional preemptive DLIs in escalating doses with a minimum interval of 3 months between DLIs. Patients with insufficient response despite multiple DLIs could receive interferon treatment.

### BM chimerism, absolute lymphocyte count, viral infections and definitions of clinical events

BM chimerism, absolute lymphocyte count (ALC) and viral infections were measured and defined as described previously [2]. The three chimerism categories were FDC, low MC (1–4% patient cells), and high MC (≥ 5% patient cells). The three ALC categories were ALC < 500 × 10^6^/l, ALC between 500 and 999 × 10^6^/l and ALC ≥ 1000 × 10^6^/l. All viral infections confirmed by PCR that occurred within 1 week before and 2 weeks after DLI without any prior relapse, second DLI or tIS were considered. Relapse was defined as recurrence of at least 5% blasts on cytomorphologic BM examination, at least 1% blasts in the peripheral blood or the development of extramedullary disease. Clinically relevant GvHD was defined as GvHD for which tIS was administered for at least 14 days [2]. 

### Analyses

Chimerism response after DLI was evaluated as described previously [19]. Briefly, an algorithm was used to assess the BM chimerism response after DLI in all patients who had MC at the time of their first DLI. A complete response was defined as conversion to FDC, and a partial response as a relative decrease in patient chimerism of 50% or an absolute decrease of 20%, 10% or 5% depending on the level of patient chimerism at the time of first DLI: ≥50%, 20–50% or < 20% MC.

The cumulative incidence of clinically relevant GvHD was calculated using a competing risks model starting at the time of first DLI with start of tIS as event of interest and relapse and death as competing events.

The current GvHD-relapse free survival (cGRFS) was calculated using two time-inhomogeneous Markov multi-state models starting at time of alloSCT (total cohort, Supplemental Fig. 1) or first DLI (DLI cohort, Supplemental Fig. 2). cGRFS was introduced by Solomon et al. and takes into account that patients can recover from GvHD, providing a more accurate measure of long-term treatment success than the GvHD-relapse free survival [20]. However, the cGRFS defined by Solomon et al. only considers moderate-severe chronic GvHD. To get insight in the total burden of clinically relevant GvHD, we considered the use of tIS for any GvHD instead [21]. 

In a multi-state model, patients move between states at the occurrence of clinical events. In the absence of relapse, patients could move between the states ‘tIS for GvHD’ and ‘cGRFS’ based on whether and when they used tIS for GvHD. From both states, patients could move to the states ‘relapse’ at time of relapse and ‘non-relapse mortality’ at time of death without relapse. The ‘relapse’ and ‘non-relapse mortality’ states were absorbing, meaning that patients could never leave these states; the probabilities of these two states represent the respective cumulative incidences. As long as no event occurred, patients remained in their current state until end of follow-up.

All analyses were performed in R version 4.4.0 using the packages prodlim [22], mstate [23], ggplot2 [24], ggalluvial [25] and ComplexUpset [26].

## Results

### Cohort

108 patients were included in this study. At 2 years after alloSCT, the cGRFS was 66% (95%-confidence interval [95%-CI] 57–77) and the cumulative incidences of relapse and non-relapse mortality were 22% (95%-CI 15–33) and 7% (95%-CI 3–13), respectively (Supplemental Fig. 3). 83 patients were included in the DLI analyses: 37 received the low-dose 4-month DLI and 46 the 6-month DLI as first DLI (Table 1). The other 25 patients did not receive a standard DLI because of early relapse (*n* = 9), GvHD (*n* = 7, of whom 6 required tIS), death without relapse or tIS (*n* = 4), or (temporary) donor unavailability (*n* = 5).

**Table 1:** Baseline characteristics of the 83 patients in the DLI cohort

	DLI cohort (*N* = 83)
Age at the time of first DLI (years)	
median (range)	60 (20–77)
Sex	
Male	50 (60%)
Female	33 (40%)
Disease	
AML^a^ 63 (76%)	
ELN adverse risk	34
ELN intermediate risk	15
ELN favourable risk (reason alloSCT: MRD+, no CR after first remission induction course, MRD+ after 2 remission induction courses)	9
relapsed AML	5
ALL	11 (13%)
B-ALL with t(9;22)	3
B-ALL, NOS	5
T-ALL	3
MDS-EB2	9 (11%)
Conditioning	
MAC: thiotepa, Flu and Bu	19 (23%)
MAC: Cy and TBI	1 (1%)
RIC: Flu, Cy and TBI	63 (76%)
Interval between stop GvHD prophylaxis and first DLI (days)	
4-month DLI patients: median (range)	33 (15–89)
6-month DLI patients: median (range)	71 (33–145)
Donor	
10/10 HLA-matched RD	15 (18%)
10/10 HLA-matched UD	50 (60%)
9/10 HLA-matched UD	17 (20%)
8/10 HLA-matched UD	1 (1%)
CMV serostatus patient/donor	
+/+	34 (41%)
+/-	9 (11%)
-/+	7 (8%)
-/-	33 (40%)
EBV serostatus patient/donor	
+/+	67 (81%)
+/-	8 (10%)
-/+	4 (5%)
-/-	4 (5%)

*DLI* donor lymphocyte infusion, *AML* acute myeloid leukaemia, *alloSCT* allogeneic stem cell transplantation, *MRD* minimal residual disease, *ALL* acute lymphoblastic leukaemia, *MDS-EB2* myelodysplastic syndrome with excess blasts, *MAC* myeloablative conditioning, *RIC* reduced-intensity conditioning, *Flu* fludarabine, *Bu* busulfan, *Cy* cyclophosphamide, *TBI* total body irradiation, *RD* related donor, *UD* unrelated donor, *CMV* cytomegalovirus, *EBV *Epstein-Barr virus

^a^AML risk scores are based on the 2022 ELN risk classification

**Fig. 1:**
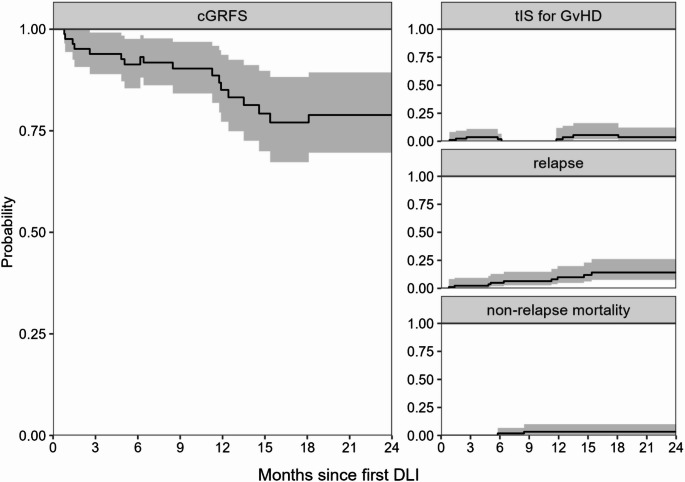
Probability of cGRFS, current use of tIS for GvHD, relapse and non-relapse mortality for all patients receiving DLI (*n* = 83). Outcome of the multi-state model over time since first DLI. The ‘relapse’ and ‘non-relapse mortality’ states are absorbing: these curves represent cumulative incidences. The structure of the model is shown in Supplemental Figure 2

For the total DLI cohort, the cGRFS was 79% (95%-CI 70–89%) at 2 years after the first DLI. At this time, the probability of using tIS was 4% (95%-CI 1−12) and the cumulative incidences of relapse and non-relapse mortality were 14% (95%-CI 8–26%) and 3% (95%-CI 1–10%), respectively (Fig. 1).

### Conditions at time of DLI

First, we examined the risk factors for GvHD that we had identified previously in the setting of DLI after alemtuzumab-based TCD: viral infections, BM chimerism (as measure for patient-derived APCs), and lymphopenia at the time of first DLI (Table 2, Supplemental Table 1). Four patients (5%) had viral infections during the week before or first two weeks after DLI. 55 patients (66%) had FDC at the time of DLI and only 5 (6%) had MC with ≥ 5% patient cells. Minimum ALC at the time of DLI was 477 × 10^6^ cells/l; 17% of the patients had lymphopenia of < 1000 × 10^6^ lymphocytes/l.

**Table 2:** Presence of viral infections, mixed BM chimerism and lymphopenia at the time of first DLI

	4-month DLI (*N* = 37)	6-month DLI (*N* = 46)
Viral infection within 1 week before until 2 weeks after DLI		
Yes	5%	4%
No	95%	96%
BM chimerism		
High mixed chimerism: ≥5% patient cells	14%	0%
Low mixed chimerism: 1–4% patient cells	32%	24%
Full donor: <1% patient cells	54%	76%
Absolute lymphocyte count		
<500 × 10^6^/l	3%	0%
500–999 × 10^6^/l	16%	15%
≥1000 × 10^6^/l	81%	85%

*DLI* donor lymphocyte infusion, *BM* bone marrow

### Alloreactivity after DLI

We then investigated the development of GvHD after DLI. Only 5 patients developed clinically relevant GvHD after DLI, resulting in a cumulative incidence of 7% (95%-CI 1–14%) at 2 years after the first DLI. None of the 5 GvHD patients had lymphopenia or a viral infection close to DLI (Supplemental Table 2). Two patients had mixed BM chimerism, 1% and 14% patient cells, at the time of their 6-month DLI. The latter had also received a 4-month DLI while having 12% MC, but did not have any GvHD symptoms until 1 month after the 6-month DLI, after which grade 4 acute GvHD developed. Despite tIS including prednisone and ruxolitinib, this patient died from GvHD 4 months after the 6-month DLI. The other three patients developed GvHD after receiving a DLI from an UD, of whom two with a 9/10 HLA-matched donor.

**Fig. 2:**
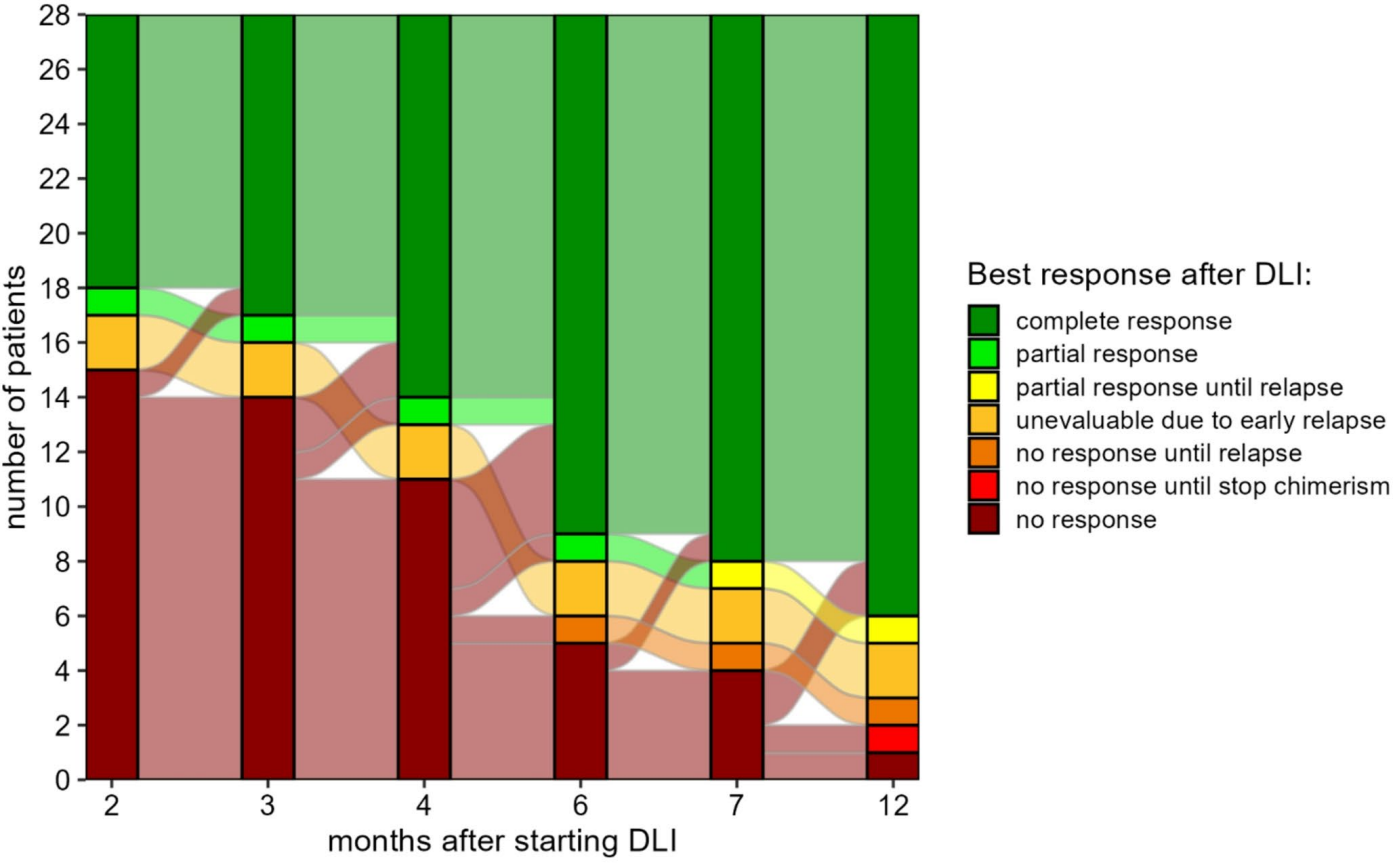
BM chimerism response after DLI for the patients with mixed chimerism at the time of first DLI (*n* = 28).The best BM chimerism response achieved at different time points after the first DLI (complete response: conversion to full-donor chimerism, partial response: decreasing mixed chimerism, no response: stable/increasing mixed chimerism). Two patients relapsed before the first chimerism measurement after DLI (relapse at 0.8 and 1.4 months after low-dose 4-month DLI) and two patients relapsed before reaching a complete response (relapse at 4.8 and 6.4 months after low-dose 4-month DLI, both also received the 6-month DLI before relapse). One other patient converted to full-donor chimerism after start of interferon. Events after reaching a complete response are not shown

To investigate whether DLI could induce conversion from MC to FDC, we examined the BM chimerism kinetics of the subset of patients with ≥ 1% MC at the time of DLI during the first year after DLI (*n* = 28, Fig. 2). 22 patients (79%) converted to FDC, including the two patients with MC who developed clinically relevant GvHD. One of the other complete responders received interferon before conversion. There were no relapses or deaths during follow-up in the complete responders except the patient with lethal GvHD (median follow-up since their first DLI: 15 months, interquartile range 12–20). Six patients did not convert to FDC: 4 relapsed and 2 did not relapse before censoring at 14 months after the first DLI. Notably, only 3 of 17 patients receiving the 4-month DLI converted before the 6-month DLI was administered. Together, these data show a low risk of GvHD following DLI in this transplantation setting (one case of lethal GvHD), but indicate achievement of a meaningful GvL effect in the majority of the patients.

## Discussion

The low risk of clinically relevant GvHD after DLI following PTCY alloSCT from HLA-matched and HLA-mismatched donors observed in our study and by others [15, 16] shows that application of non-haploidentical DLI after PTCY is relatively safe. The 4% cumulative incidence at 3 months is strikingly lower than the 30% we observed after DLI following alemtuzumab-based TCD alloSCT [2]. Against the background of our previous study [2], infrequent occurrence of DLI-induced GvHD after non-haploidentical PTCY alloSCT can be explained by the relatively high prevalence of FDC at the time of DLI, absence of deep lymphopenia, and low incidence of viral infections around the time of DLI. A combined interpretation of the results of this study and our study on DLI after alemtuzumab-based TCD [2] indicates that transplantation strategies have a profound impact on the conditions at the time of DLI, which in turn influence the alloreactive potential of DLI. The impact of the conditioning regimen on DLI alloreactivity was also noted by Shanmugasundaram et al., who observed GvHD only in patients who received alemtuzumab or anti-thymocyte globulin in addition to the PTCY [16]. 

In both our studies [2], none of the FDC patients receiving DLI developed lethal GvHD. Together with the results of a matched-pair analysis by Schmid et al. [27], this demonstrates the safety of prophylactic non-haploidentical DLI. The patient with high MC developing lethal DLI-induced GvHD illustrates the relevance of high presence of patient-derived APCs and shows that under certain conditions DLI after PTCY can induce lethal GvHD. Of the other patients developing GvHD after DLI, 2 had an HLA mismatch. While PTCY may reduce the impact of having an HLA mismatch on the GvHD risk after non-haploidentical alloSCT [28], this effect is likely smaller when fresh alloreactive lymphocytes are infused by a DLI several months thereafter when the degree of genetic disparity may play an important role in the development of GvHD. The low number of GvHD cases in our cohort did not allow us to estimate the effect sizes of mixed chimerism and HLA mismatch on the risk of GvHD.

The low-dose 4-month DLI after PTCY alloSCT rarely induced chimerism conversion or clinically relevant GvHD, suggesting limited alloreactive potential in contrast to the 3-month low-dose DLI after alemtuzumab-based TCD alloSCT [2, 19]. This is likely due to the different conditions at the time of DLI: the faster lymphocyte recovery after PTCY alloSCT compared to alemtuzumab-based TCD leads to less viral infections and therefore less inflammation during the months after alloSCT. Combined with the later timing of the low-dose DLI after PTCY alloSCT (one month later than after alemtuzumab-based TCD alloSCT), this leads to a less pro-inflammatory environment at the time of DLI and a low alloreactive potential of the 4-month DLI after PTCY alloSCT with the current DLI dose. However, the total DLI strategy led to similar conversion rates for DLI after alemtuzumab or PTCY [19]. In both settings [19], conversion from MC to FDC after DLI occurred often in the absence of clinically relevant GvHD, but the GvHD/GvL balance seems to be better in the PTCY setting: the doses of the 6-month DLI and any subsequent DLI were apparently sufficient to induce chimerism conversion, but with a lower GvHD risk than in the alemtuzumab setting. This supports the conclusions of Van Bergen et al. that whether or not GvL is companied with GvHD not only depends on the diversity of the alloreactive T cells but also on the inflammatory conditions [29]. Differences in the timing and doses of DLI and the conditions at the time of infusion might explain why the chimerism conversion rates in our studies differ from those reported by Shanmugasundaram et al. [16]. 

The aim of prophylactic and preemptive DLI is to prevent relapse without causing excessive toxicity. With our total strategy of TCD alloSCT followed by standard DLI, the 2-year cumulative incidence of relapse was 22%. This is still close to the estimates reported in studies on non-haploidentical PTCY alloSCT for acute leukaemia without DLI, which range from 19% in a single-centre study to 28% in a 9/10 HLA-matched UD registry cohort [28, 30, 31]. The 2-year non-relapse mortality in our study (7%) seems be a bit lower than in the other studies (15–20%) [28, 30, 31]. Comparing studies is notoriously difficult because of differences in transplantation strategy and characteristics of the patients, diseases and donors. However, in our study none of the patients who converted to FDC after DLI experienced relapse, indicating that a meaningful GvL effect was achieved. Together with the low toxicity, this strongly suggests that application of DLI after non-haploidentical PTCY alloSCT can have a beneficial clinical effect. In our cohort, about half of the relapsing patients relapsed between 3 and 6 months after alloSCT. Considering the low toxicity and efficacy of our 4-month low-dose DLI, it might be possible to increase the dose of this DLI or to administer the current dose at an earlier time to reduce the relapse risk during this period without inducing severe GvHD.

A limitation of our study is that we do not have a control group of patients not receiving standard DLI. Since most alloreactivity was observed after the 6-month DLI, several months after cessation of double GvHD prophylaxis, we assume that the observed alloreactivity is DLI-induced, but cannot rule out some effect of the tapering of GvHD prophylaxis. However, after PTCY alloSCT combined with double GvHD prophylaxis using HLA-mismatched donors and no DLI, Soltermann et al. observed a cumulative incidence of only 15% acute GvHD grade II-IV, predominantly occurring during the first 2 months [32]. This suggests that the GvHD we observed after the 4- and 6-month DLI is most likely related to the DLI. We are currently planning a clinical trial to investigate the optimal timing and dose of prophylactic DLI and to compare alloSCT with or without standard prophylactic DLI.

In conclusion, our data show that non-haploidentical prophylactic and preemptive DLI following PTCY alloSCT give a low risk of clinically relevant GvHD but still a meaningful GvL effect. The conditions in which DLI are more likely to induce severe GvHD are known. Careful tailoring the DLI dose to the conditions at the time of the DLI could therefore improve the balance between GvHD and GvL and increase the safety and efficacy of DLI.

## Supplementary Information

Below is the link to the electronic supplementary material.

Supplementary Material 1 (PDF 297 KB)

## Data Availability

The data that support the findings of this study are available from the corresponding author upon reasonable request.
