# Supplementary material for: Transplantation strategy affects the risk of GvHD after prophylactic and preemptive donor lymphocyte infusion
Source: Ann Hematol. 2025 Oct 20;104(10):5379–87. doi: 10.1007/s00277-025-06662-x (PMC12619706; doi:10.1007/s00277-025-06662-x)
Supplement: Supplementary file 1 — Supplementary Material 1 (PDF 297 KB) [file 277_2025_6662_MOESM1_ESM.pdf]

Supplemental Tables

Supplemental Table 1: Numbers of patients for each combination of characteristics of BM chimerism, ALC and viral infection

BM, bone marrow; FDC, full-donor chimerism; MC, mixed chimerism; ALC, absolute lymphocyte count (x10^6/l); DLI, donor lymphocyte infusion

		BM chimerism: FDC	BM chimerism: 1-4% MC	BM chimerism: ≥5% MC
**No viral infection close to DLI**[a]	**ALC ≥1000**	43	18	4
	**ALC 500-999**	10	2	1
	**ALC <500**	0	1	0
**Viral infection close to DLI**[a]	**ALC ≥1000**	2 (both COVID-19)	2 (CMV, rhinovirus)	0
	**ALC 500-999**	0	0	0
	**ALC <500**	0	0	0

[a]Within 1 week before until 2 weeks after DLI

Supplemental Table 2. Characteristics of the 5 patients who developed GvHD after DLI

MM, HLA-mismatched (else: 10/10 HLA-matched); RD, related donor; UD, unrelated donor; BM, bone marrow; FDC, full-donor chimerism; MC, mixed chimerism; ALC, absolute lymphocyte count; aGvHD, acute GvHD; cGvHD, chronic GvHD; tIS, therapeutic systemic immunosuppression

Donor	Last DLI before onset of GvHD	BM chimerism at time of DLI	ALC at time of DLI	Viral infection close to DLI	GvHD requiring tIS[a]	Outcome
MM UD	4-month DLI	FDC	≥1000	No	• aGVHD liver grade 1<br>• extensive cGVHD liver, muscles	Resolved
MM UD	6-month DLI after 4-month DLI	FDC	≥1000	No	• extensive cGVHD eyes, nails	Ongoing tIS 1 year after DLI
UD	6-month DLI	FDC	≥1000	No	• Extensive cGVHD lungs, muscles	Ongoing tIS 1 year after DLI
RD	6-month DLI	1% MC	≥1000	No	• Extensive cGvHD skin	Ongoing tIS 9 months after DLI
RD	6-month DLI after 4-month DLI	12% MC	≥1000	No	• aGVHD skin, liver, GI grade 4	Death from GvHD

[a]Grading of acute and chronic GvHD according to the modified Glucksberg and the Seattle criteria, respectively.

Supplemental Figures

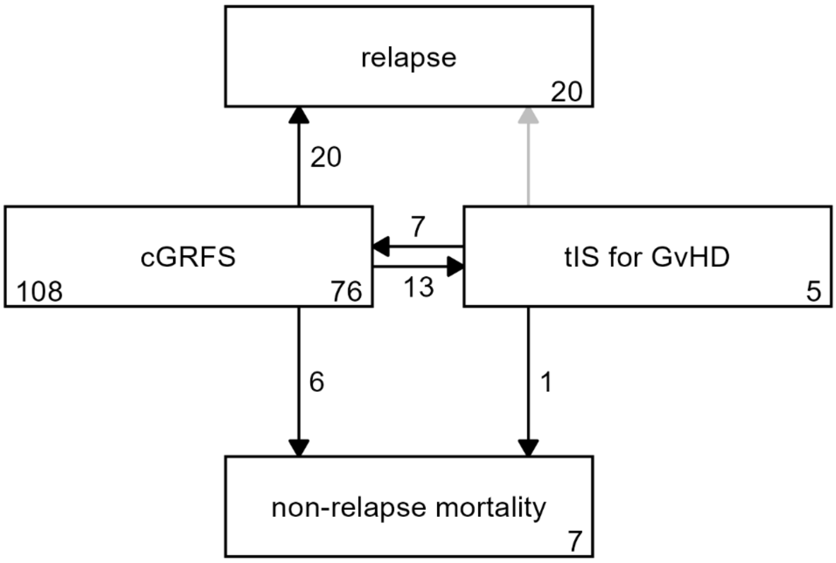

Supplemental Figure 1. Multi-state model starting from alloSCT (total cohort). Boxes represent states and arrows represent the transitions between the states. The grey transition was not used by any of the included patients. All patients started in the state 'cGRFS' at the time of alloSCT. The number at the bottom left corner of the starting state shows the number of patients included in the model. The numbers at the bottom right corner of the boxes show the numbers of the patients who were in that state at the end of their follow-up. The numbers next to the arrows show the numbers of the patients who made that transition during their follow-up.

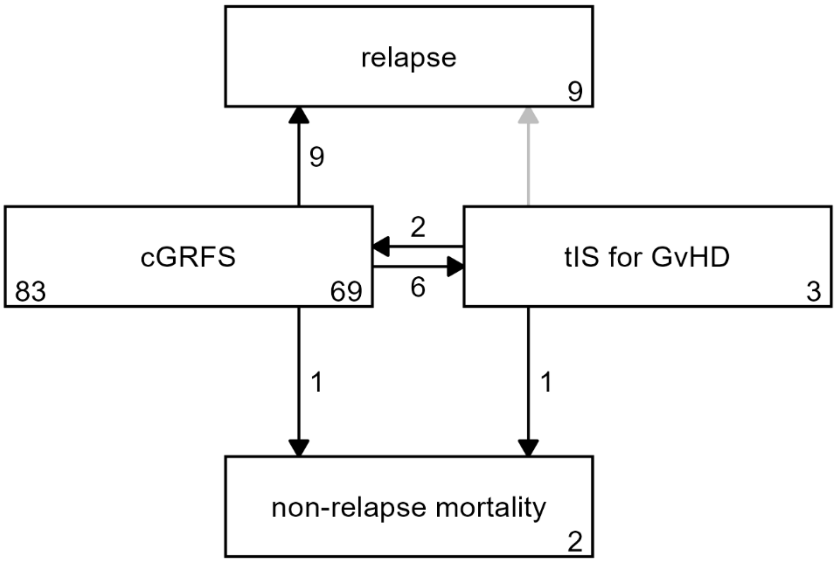

Supplemental Figure 2. Multi-state model starting from first DLI (DLI cohort). Boxes represent states and arrows represent the transitions between the states. The grey transition was not used by any of the included patients. All patients started in the state 'cGRFS' at the time of their first DLI. The number at the bottom left corner of the starting state shows the number of patients included in the model. The numbers at the bottom right corner of the boxes show the numbers of the patients who were in that state at the end of their follow-up. The numbers next to the arrows show the numbers of the patients who made that transition during their follow-up.

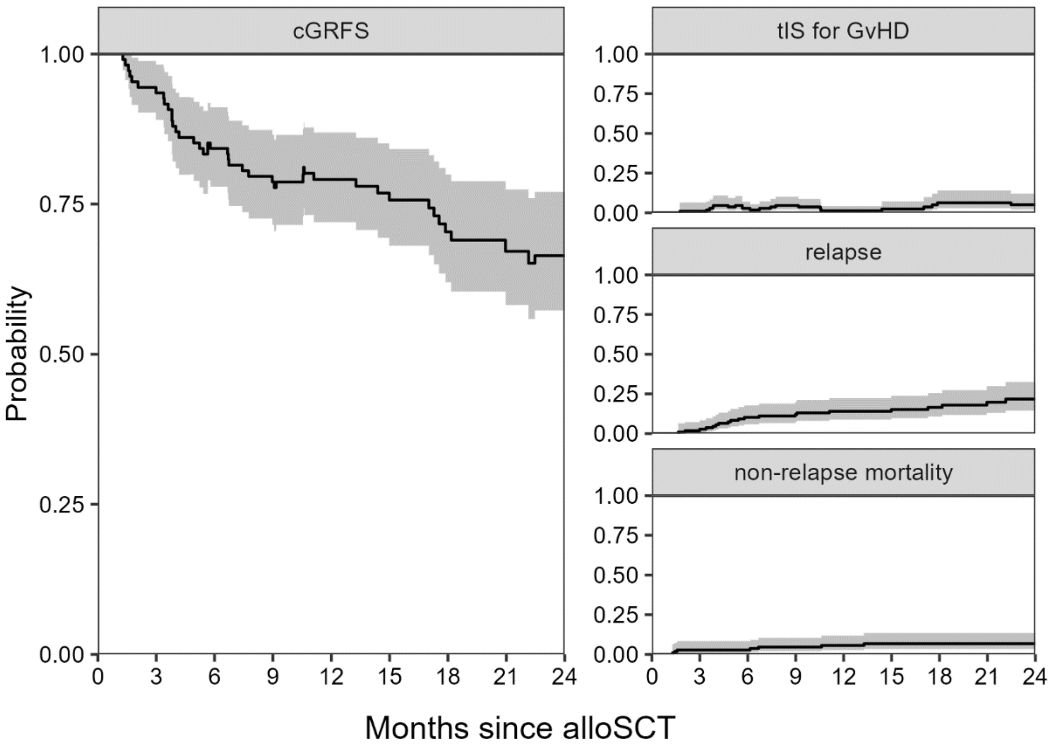

Supplemental Figure 3. Probability of cGRFS, current use of tIS for GvHD, relapse and non-relapse mortality after alloSCT (total cohort). The ‘relapse’ and ‘non-relapse mortality’ states are absorbing: these curves represent cumulative incidences. The structure of the model is shown in **Supplemental Figure 1**.
